# Missed diagnostic opportunities within South Africa’s early infant diagnosis program, 2010–2015

**DOI:** 10.1371/journal.pone.0177173

**Published:** 2017-05-11

**Authors:** Ahmad Haeri Mazanderani, Faith Moyo, Gayle G. Sherman

**Affiliations:** 1 Centre for HIV & STIs, National Institute for Communicable Diseases, Johannesburg, South Africa; 2 Department of Medical Virology, University of Pretoria, Pretoria, South Africa; 3 Paediatric HIV Diagnostic Syndicate, Wits Health Consortium, Johannesburg, South Africa; 4 Department of Paediatrics & Child Health, Faculty of Health Sciences, University of the Witwatersrand, Johannesburg, South Africa; Waseda University, JAPAN

## Abstract

**Background:**

Samples submitted for HIV PCR testing that fail to yield a positive or negative result represent missed diagnostic opportunities. We describe HIV PCR test rejections and indeterminate results, and the associated delay in diagnosis, within South Africa’s early infant diagnosis (EID) program from 2010 to 2015.

**Methods:**

HIV PCR test data from January 2010 to December 2015 were extracted from the National Health Laboratory Service Corporate Data Warehouse, a central data repository of all registered test-sets within the public health sector in South Africa, by laboratory number, result, date, facility, and testing laboratory. Samples that failed to yield either a positive or negative result were categorized according to the rejection code on the laboratory information system, and descriptive analysis performed using Microsoft Excel. Delay in diagnosis was calculated for patients who had a missed diagnostic opportunity registered between January 2013 and December 2015 by means of a patient linking-algorithm employing demographic details.

**Results:**

Between 2010 and 2015, 2 178 582 samples were registered for HIV PCR testing of which 6.2% (n = 134 339) failed to yield either a positive or negative result, decreasing proportionally from 7.0% (n = 20 556) in 2010 to 4.4% (n = 21 388) in 2015 (p<0.001). Amongst 76 972 coded missed diagnostic opportunities, 49 585 (64.4%) were a result of pre-analytical error and 27 387 (35.6%) analytical error. Amongst 49 694 patients searched for follow-up results, 16 895 (34.0%) had at least one subsequent HIV PCR test registered after a median of 29 days (IQR: 13–57), of which 8.4% tested positive compared with 3.6% of all samples submitted for the same period.

**Conclusions:**

Routine laboratory data provides the opportunity for near real-time surveillance and quality improvement within the EID program. Delay in diagnosis and wastage of resources associated with missed diagnostic opportunities must be addressed and infants actively followed-up as South Africa works towards elimination of mother-to-child transmission.

## Introduction

In the absence of antiretroviral therapy (ART), HIV infection during infancy is associated with rapid disease progression with more than half of all infected children expected to die before two years of age [1,2]. Furthermore, data from South Africa suggests a peak in infant mortality occurring as early as two to three months of age. Fortunately, early initiation of ART has been found to markedly reduce HIV-associated morbidity and mortality [3], thereby highlighting the need for early diagnosis and linkage to care [4–6]. However, on account of the passive transfer of maternally acquired anti-HIV antibodies, early infant diagnosis (EID) of HIV requires direct virological methods that are distinct from standard serological assays used for adult testing [7]. Although expensive, costing up to ten-times more than antibody tests [8], the World Health Organisation (WHO) nevertheless recommends HIV-exposed infants undergo virological testing, such as polymerase chain reaction (PCR) tests, at birth and 6 weeks of age [7].

South Africa’s EID program has evolved considerably from when it was launched a decade ago. Whereas testing guidelines in 2004 recommended a single PCR test at 6 weeks of age, current recommendations include testing at birth, 10 weeks of age and 6 weeks post-cessation of breastfeeding; with an additional HIV PCR confirmatory test performed for those infants who test positive [9–12]. Testing is routinely performed on whole blood specimens collected either on dried blood spot (DBS) cards by means of capillary heel-prick or EDTA anticoagulated blood via phlebotomy, with the former associated with improvement in testing coverage [13,14]. With South Africa’s antenatal HIV prevalence remaining around 30% since 2004, the volume of samples submitted within the EID program has increased dramatically over the years, from 13 069 HIV PCR tests registered in 2004, to 294 730 in 2010, and 485 458 in 2015 [13,15]. Yet despite this, HIV PCR testing is still performed at centralized facilities, with only nine EID laboratories currently operating within South Africa’s National Health Laboratory Service (NHLS), the only diagnostic laboratory service within the public health sector.

Although different HIV PCR assays, utilising separate standard operating procedures and laboratory information systems (LIS) have been used since EID testing became available, better standardised laboratory practices have been introduced in recent years. Since 2010, all EID laboratories within the public sector have utilized the same assay, the COBAS® AmpliPrep/COBAS® TaqMan (CAP/CTM) HIV-1 Qualitative Test (Roche Molecular Systems, Inc., Branchburg, NJ); with a newer version, the CAP/CTM v2.0, with a lower limit of detection, introduced during the course of 2014 [16,17]. Furthermore, all national EID facilities, which are accredited diagnostic laboratories (ISO 15189:2012), have adopted a single standard operating procedure that outlines testing procedures and interpretation of results, including criteria to distinguish indeterminate from positive results [18,19]. Standardized reporting practices have been further bolstered due to successful implementation of a single LIS throughout the NHLS, a process that began in 2010 and was finalized in 2015.

The increased uniformity of EID laboratory practice within South Africa has greatly facilitated surveillance efforts, with the value of routine laboratory data for monitoring mother-to-child transmission rates and infant testing coverage well described [13,14]. On account of an effective prevention of mother-to-child transmission (PMTCT) program (progressing from WHO Option A to Option B in 2013 and Option B+ in 2015), laboratory data has demonstrated a dramatic decline in the early infant transmission rate from 17.0% in 2005, to 4.3% in 2010, and 1.8% in 2014 [13,20]. Nevertheless, due to the considerably high national maternal HIV prevalence there remain a substantial number of infants infected per annum, with South Africa unlikely to meet WHO impact targets for the elimination of mother-to-child transmission within the foreseeable future [21,22]. Hence, South Africa’s EID program is likely to remain an essential component of child health services over the coming years. It is therefore imperative that, in addition for the need to prioritise early diagnosis and linkage to care of HIV-infected infants, the quality of testing within the EID program be monitored and maintained.

Whereas missed opportunities for specimen collection have been described within clinical settings [23], the reasons for and extent of specimens submitted for HIV PCR testing that fail to yield either a positive or negative result have to date not been reported. Although some test-sets may be rejected on account of not requiring a result, such as registration errors, the remainder represent missed diagnostic opportunities (MDOs). These include errors that occur prior to testing as well those that arise from the testing process itself [24], both of which have important implications for patient care as well as monitoring and surveillance. Furthermore, on account of the considerable expense associated with PCR testing, MDOs need to be investigated and monitored. Understanding where errors occur within the testing process can assist with redesigning systems that render it difficult for health-care professionals to make mistakes, thereby reducing wastage of resources both within the clinic and laboratory [24]. Furthermore, due to the dramatic increase in the volume of testing performed over recent years as well as concerns that changes within the PMTCT program and testing landscape could impact negatively on diagnostic quality [25,26], assessing the trend of MDOs is imperative. Towards this end we describe HIV PCR test rejections and indeterminate results, and the associated delay in diagnosis, within South Africa’s early infant diagnosis program from 2010 to 2015.

## Methods

### Missed diagnostic opportunities

All samples registered for HIV PCR testing within South Africa’s public health sector from 01 January 2010 to 31 December 2015 that failed to yield either a positive or negative result were extracted from the NHLS Corporate Data Warehouse (CDW), the central data repository of all registered test-sets within the NHLS. Data were extracted at a single time-point by laboratory number, result, rejection code, reviewed date, facility name, and testing laboratory. Additionally, the total number of samples registered for HIV PCR testing from 2010 to 2015 were extracted from the NHLS CDW according to year, result, province and testing laboratory. On account of standardized error-codes being introduced only with the current LIS, samples rejected prior to this were extracted as non-coded errors whereas samples rejected on the current LIS were extracted as coded errors. Categorization of coded errors was performed by two pathologists who generated inductive groups, informed by the LIS rejection codes, which were then validated against the original data by a third researcher and inconsistencies resolved by consensus.

All coded-errors were subdivided according to whether a result was required or not as indicated by the rejection text. Missed diagnostic opportunities, defined as samples registered for HIV PCR testing that required but did not yield a valid positive or negative result, were further categorized as either pre-analytical or analytical error, depending on whether the sample was rejected prior to HIV PCR testing or subsequent to testing, as indicated by the rejection code (Table 1). Pre-analytical error was further broken down into healthcare worker error and pre-analytical laboratory error, with healthcare worker error categorized into three distinguishable groups, namely samples with insufficient volume, unsuitable sample type or quality, and clerical error. The group categorized as unsuitable sample type or quality involved a broad range of rejection codes including samples rejected as simply ‘poor quality’ with no further details provided on the LIS, as well as samples indicated as ‘clotted’ and ‘incorrect sample type.’ Analytical errors were also categorised into three groups, namely indeterminate results, invalid results and non-specific analytical error. Indeterminate results are defined as HIV PCR tests that yield an inconclusive result that is interpreted as being neither clearly positive nor negative [27]. Various laboratory-specific cycle threshold and relative fluorescence intensity cut-offs have been used to define this analytical grey-zone since the introduction of the EID program, with standard operating procedures being adopted throughout the NHLS in 2013 [19]. Unlike indeterminate results, invalid results fail to yield a valid instrument result after testing and can be attributed, amongst other reasons, to PCR inhibition. Non-specific analytical errors refer to samples rejected on the basis of a ‘technical problem’ or ‘non-reportable lab error’ with no further details provided.

**Table 1:** Classification of missed diagnostic opportunities by rejection code.

Type of Error	Classification	Examples of LIS Rejection Codes
**Pre-Analytical Error**	Insufficient	• Insufficient sample volume for testing • Sample container empty
	Unsuitable	• Not done: Poor quality sample Unsuitable for testing: Sample clotted • Incorrect sample type: Require EDTA sample
	Clerical Error	• Labelling error: Information on form differs from sample • Incomplete form: No age or date of birth • Incomplete form: No patient name or surname
	Pre-Analytical Lab Error	• Sample lost in transit • Sample leaked in transit • Sample too old for testing • Not done: Sample spun for plasma test
**Analytical Error**	Indeterminate	• Indeterminate result
	Invalid	• Invalid result: PCR inhibition
	Lab Error Not Specified	• Technical problem

LIS, Laboratory Information System

Both non-coded and coded error groups were included in the national error trend analysis. However, as non-coded errors could not be categorized into either pre-analytical or analytical groups they were excluded from the provincial and laboratory MDO analysis which was performed on the subset of tests reviewed between 2013 and 2015. Descriptive analysis was performed using Microsoft Excel with annual proportions of pre-analytical, analytical and non-coded error compared using Pearson’s Chi-square test in Stata version 14 (Statacorp, Texas).

### Linkage to care

In order to determine the proportion of patients with an MDO who had a subsequent linked HIV PCR test, as well as determining the results and time to repeat testing, all samples that received an HIV PCR result that was neither positive nor negative between 01 January 2013 to 31 December 2015, including both coded and non-coded errors, were extracted from the NHLS CDW. Additionally, all HIV PCR tests registered between 01 January 2013 and 30 April 2016 were extracted from the CDW. Results from these two datasets were then linked by means of an automated patient linking-algorithm using probabilistic matching employing patient demographic details including first name, surname, date of birth, and laboratory or facility number. Descriptive analysis was then performed on the linked results using Stata version 14 (Statacorp, Texas).

This study was approved by the University of Pretoria’s Faculty of Health Sciences Ethics Committee (Protocol number—41/2016).

## Results

**Fig 1:**
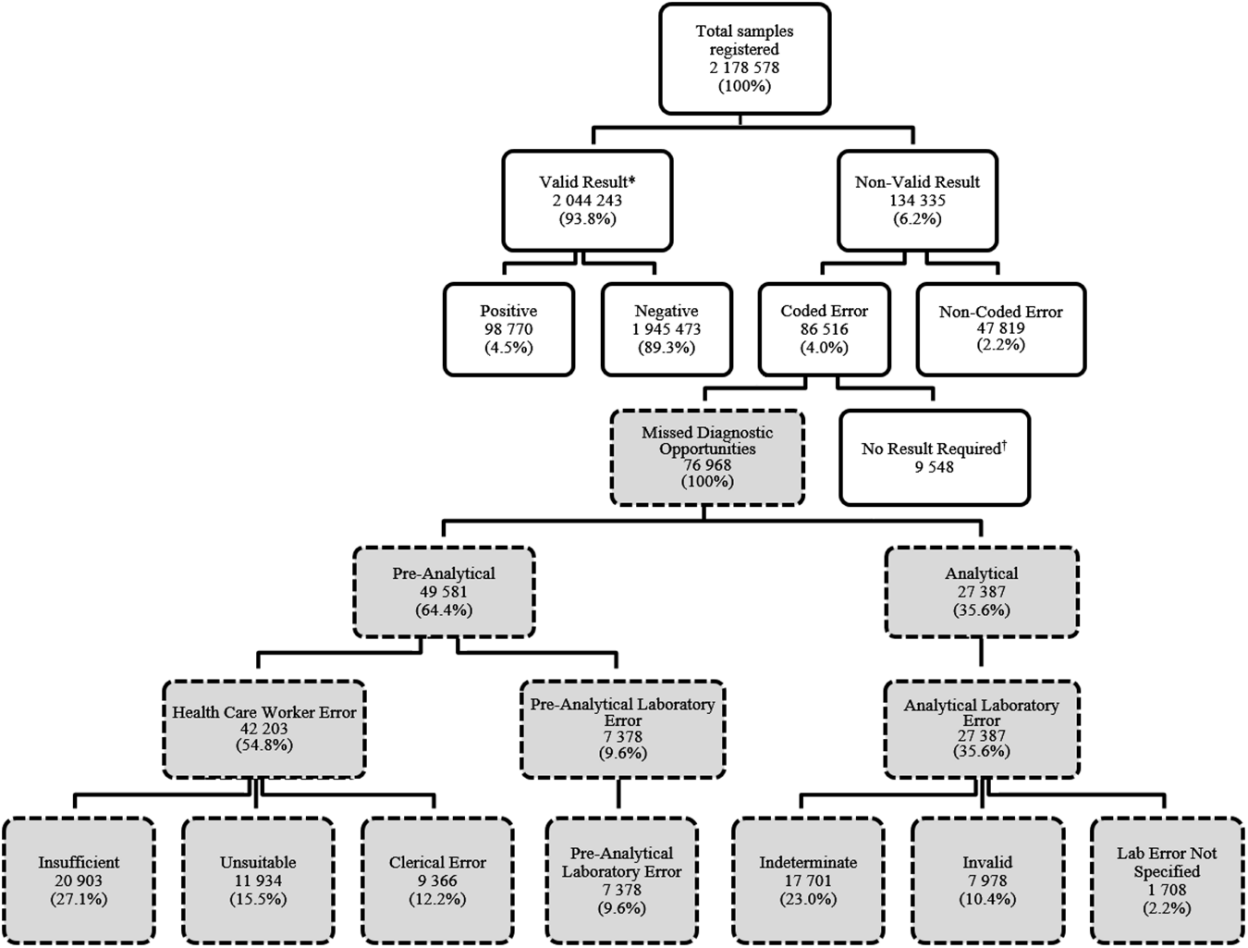
Samples registered for HIV PCR testing between 2010–2015 categorized by result or rejection code. *Results verified as indeterminate were categorized as non-valid results in this analysis. ^†^As indicated by the rejection code on the laboratory information system (e.g. duplicate registration).

**Fig 2:**
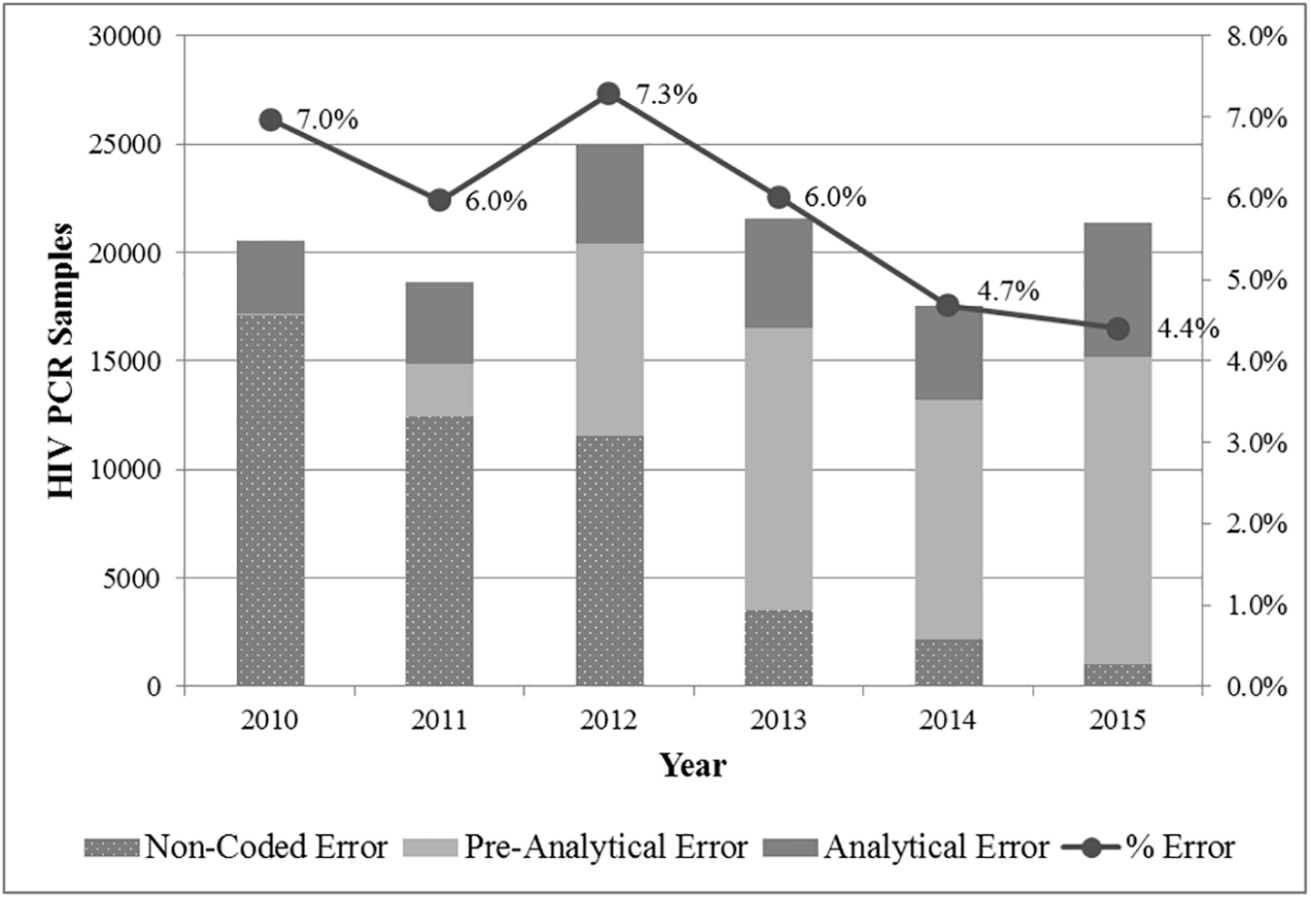
Non-coded, pre-analytical and analytical error 2010–2015.

A total of 2 178 578 samples from the South African public health sector were registered for HIV PCR testing within the NHLS from 2010 to 2015 of which 98 770 (4.5%) were resulted as positive, 1 945 473 (89.3%) negative and 134 335 (6.2%) received neither a valid positive or negative result (Fig 1). The proportion of samples that failed to yield a positive or negative result decreased significantly between 2010 and 2015 from 7.0% (n = 20 556) to 4.4% (n = 21 388) (p<0.001) (Fig 2). Of these, 47 819 were non-coded errors, declining significantly from 17 098 samples (5.8%) in 2010 to 1 017 samples (0.2%) in 2015 (p<0.001), and 86 516 were coded errors. A total of 9 548 samples were excluded from the coded error group because they were rejected on account of not requiring a result as indicated by the LIS rejection code. Examples of these include samples rejected because of duplicate registration or where HIV PCR testing was not clinically indicated. The remaining 76 972 specimens in the coded error group, defined as MDOs, were further categorized into either pre-analytical or analytical error groups.

Amongst samples identified as MDOs, pre-analytical error comprised the majority, totalling 49 585 (64.4%), whereas analytical error accounted for the remaining 27 387 (35.6%) samples. Between 2013 and 2015, 3.6% of registered samples tested positive whereas 4.9% of samples were MDOs, ranging from 2.7% to 7.3% across the nine national provinces as follows: Mpumalanga (2.7%), Free State (3.3%), North West (3.4%), Gauteng (3.5%), Limpopo (3.5%), Western Cape (4.7%), Northern Cape (5.8%), Kwa-Zulu Natal (6.7%), and Eastern Cape (7.3%).

### Pre-analytical error

Between the years 2010 and 2015, healthcare worker error comprised 85.1% of all pre-analytical error, totalling 42 203 samples of which 20 903 (49.5%) were rejected due to insufficient sample volume, 11 934 (28.3%) were unsuitable for testing on account of sample type or quality, and 9366 (22.2%) were rejected due to clerical error (Fig 1). Pre-analytical laboratory error comprised the remaining 14.9% of pre-analytical error, totalling 7 382 samples of which 5 414 (73.4%) were rejected on account of the sample or request form being lost or the sample leaking during transit, 891 (12.0%) were considered too old for processing on arrival in the testing laboratory, and the remaining 1 077 (14.6%) were rejected due to incorrect lab handling prior to testing (e.g. specimen spun for plasma testing). Pre-analytical error trends from 2013 to 2015 show a reduction in the number of rejections due to insufficient sample volume but a simultaneous increase in unsuitable samples, clerical error, and pre-analytical laboratory error (Table 2).

**Table 2:** Reason for missed diagnostic opportunities 2013–2015.

Type of Error	Classification	2013	2014	2015
Pre-Analytical Error	Insufficient sample	7 790 (43.3%)	4 019 (26.1%)	3 716 (18.2%)
	Unsuitable sample	2 045 (11.4%)	3 184 (20.7%)	3 986 (19.6%)
	Clerical error	1 478 (8.2%)	2 012 (13.1%)	3 630 (17.8%)
	Pre-analytical lab error	1 419 (7.9%)	1 578 (10.3%)	2 515 (12.3%)
Analytical Error	Indeterminate	2 890 (16.0%)	2 900 (18.8%)	3 246 (15.9%)
	Invalid	1 685 (9.4%)	1 004 (6.5%)	2 429 (11.9%)
	Lab error not specified	7 02 (3.9%)	691 (4.5%)	853 (4.2%)
Total	MDOs	18 009	15 388	20 375

### Analytical error

Between 2010 to 2015 there were 17 701 indeterminate results (64.6% of analytical error), 7 978 invalid results (29.1%), and 2 761 non-specific laboratory errors (6.3%). Although indeterminate results comprised only 0.8% of all HIV PCR tests during this period, they represent 15.2% of all non-negative valid results (i.e. positive and indeterminate results combined). Between 2013 and 2015 the proportion of indeterminate results (using non-negative valid results as a denominator) fluctuated minimally from 17.2% in 2013, 17.6% in 2014, and 16.9% in 2015, although the absolute number increased from 2 890 samples in 2013 (0.8% of all HIV PCR requests) to 2 900 samples in 2014 (0.8%), and 3 246 samples in 2015 (0.7%) (Table 2). Invalid results were found to decrease from 1 685 samples in 2013 (0.5%) to 1 004 samples in 2014 (0.3%), and then subsequently increase to 2 429 samples in 2015 (0.5%). A breakdown of analytical error per testing laboratory during this period demonstrates that indeterminate results represent the majority of analytical error in all except one laboratory, with this same laboratory contributing 83.9% of all invalid results.

### Linkage to care

Between 2013 and 2015, a total of 55 035 samples belonging to 49 694 patients met the inclusion criteria to determine follow-up characteristics of patients who received an MDO result. 16 895 patients (34.0%) were found to have a subsequent HIV PCR test, registered after a median of 29 days with an inter quartile range (IQR) of 13–57 days, of which 13 302 (78.7%) were negative, 1 415 (8.4%) were positive, and 2 178 (12.9%) received a second result which was neither positive nor negative. Amongst the 1 415 patients who subsequently tested positive, the median time to follow-up testing was 28 days (IQR 7–63 days). Of the error codes associated with the initial MDO, 578 (40.8%) were due to pre-analytical error, 714 (50.5%) analytical error (637 of which were indeterminate results), and the remaining 123 (8.7%) were due to a mixture of non-coded errors and other reasons, such as duplicate registration. There were 605 (42.8%) patients who tested positive on two separate occasions after receiving an initial MDO result, with a median time from the first MDO to the confirmatory PCR result of 84 days (IQR 32–169). In these cases, the MDO delayed confirmation of an HIV-infected status from a median of 28 to a median of 84 days.

## Discussion

Despite a considerable increase in the total number of samples submitted for testing within the EID program between 2010 and 2015, the total number of registered HIV PCR tests that failed to yield either a positive or negative result increased by only 832 samples. Expressed as a percentage, ‘total errors’ actually decreased from 7.0% to 4.4%. Amongst samples defined as MDOs, 64.4% were rejected prior to testing, in keeping with data that suggest pre-analytical error accounts for the majority of mistakes that occur in laboratory medicine [28]. Most of these were attributable to mishandling procedures during collection, such as samples submitted with insufficient volume, labelling errors, and incorrect sample type (e.g. incorrect collection tube) and clotting.

Because of the large number of non-coded errors, where samples were rejected but the rejection reason was either not provided or could not be retrieved from the LIS, it is not possible to accurately describe pre-analytical and analytical rejection trends between 2010 and 2015. However, over this period there has been a significant reduction in the number of these non-coded errors which can be attributed to the introduction of a single LIS throughout the NHLS. Standardised rejection practices have in turn facilitated the use of laboratory data for surveillance purposes. The reduction in the number of insufficient samples between 2013 and 2015 has occurred in parallel with work done by NHLS EID trainers using NHLS CDW data for identifying facilities with high rates of insufficient samples and conducting in-service training to address this problem (Table 2).

Despite concerns that changes both in PMTCT guidelines and diagnostic practices introduced over recent years could result in increased analytical error, this has not been observed at a national level. For example, the increased exposure to ART prophylaxis amongst infants, associated with WHO Option B/B+, as well as the introduction of routine birth testing within the EID programme have both been posited as potential contributing factors towards indeterminate and invalid HIV PCR results [25]. However, the proportion of indeterminate results has remained fairly constant since 2011. Invalid results, on the other hand, have fluctuated, increasing between 2014 and 2015. On further investigation it was found that this was due to rejection practices at a single high-throughput laboratory which had stopped the standard practice of repeat-testing prior to verification of samples that yielded invalid results. This was brought to the attention of the laboratory and corrective action taken, further illustrating the potential utility of routine laboratory data and benefit of proactive monitoring.

Regardless of whether a sample fails to yield a valid result on account of pre-analytical or analytical error, follow-up remains essential. With only one third of patients with an MDO having evidence of repeat testing, our findings suggest that in the majority of such cases the opportunity for an early HIV result is missed. This is all the more important considering the positivity rate amongst patients with a previous MDO (8.4%) was found to be much higher than the positivity rate amongst total samples submitted for HIV PCR testing (3.6%) during the same period. Pre-analytical and analytical reasons, other than indeterminate results, were found to comprise the majority of these cases suggesting it is not just patients with indeterminate results that require close follow-up but rather all MDOs. It was also found that less than half of the patients with an MDO who subsequently tested positive had evidence of a confirmatory test, suggesting poor linkage into care. Furthermore, the delay in diagnosis from when the initial specimen was registered to when the confirmatory test was registered amounted to a median delay correlating with the peak mortality rate amongst HIV-infected infants in South Africa of two to three months [4]. Patients with an MDO therefore represent an at-risk group that could benefit from active follow-up, second only to infants who test positive.

There are a number of important limitations to consider regarding this study as well as areas where further analysis is required. Although routine birth testing, which was introduced into national guidelines in June 2015, does not appear to be associated with an immediate upsurge of either pre-analytical or analytical error, monitoring is ongoing. Similarly, data was not available to determine whether there was an association between sample type (i.e. DBS or EDTA-anticoagulated whole blood) and particular MDOs. Additional limitations relate to the patient linking-algorithm, which has a reported sensitivity of only 73% and positive predictive value of 83% in adult patients [29]. Hence, on account of transcription and data-capturing errors, as well infants registered under their mother’s details and name-changes that may occur during early childhood, the true follow-up rate of patients could not be determined. The patient linking-algorithm employed in this study provides at best a conservative estimate of patient follow-up and is likely an under-estimate of the true follow-up rate. Until South Africa employs a unique patient identifier, this problem will remain an inherent limitation of using laboratory data from the public sector.

## Conclusions

Routine laboratory data provides the opportunity for near real-time surveillance and quality improvement within the EID program. Despite a decrease in the proportion of HIV PCR test-sets that failed to yield either a positive or negative result between 2010 and 2015, there remain unacceptably high volumes of rejected and indeterminate samples within South Africa’s infant testing program. Whilst it is understood that complete elimination of laboratory testing error is unrealistic, improved communication among caregivers is considered a practical means of reducing the HIV PCR rejection rate [30]. Towards this end, monthly reports are being distributed to clinics and laboratories specifying the number and reasons for MDOs. It is hoped that near real-time feedback of a simplified indicator and multilevel leadership support will lead to the necessary quality improvements within the national EID program, thereby facilitating a timely and definitive diagnosis for all HIV-exposed infants in South Africa [14,31]. Essentially, the delay in diagnosis and wastage of resources associated with rejected and indeterminate results must be addressed and infants actively followed-up as South Africa works towards eliminating mother-to-child transmission of HIV.

## Supporting information

S1 File National MDO data 2010–2015. MDO, Missed Diagnostic Opportunities.
(ZIP)
